# An Agent-Based Model to study the epidemiological and evolutionary dynamics of Influenza viruses

**DOI:** 10.1186/1471-2105-12-87

**Published:** 2011-03-30

**Authors:** Benjamin Roche, John M Drake, Pejman Rohani

**Affiliations:** 1 Department of Ecology and Evolutionary Biology, University of Michigan, Ann Arbor, MI 48109, USA; 2 UMI IRD/UPMC 209 - UMMISCO, 93143, Bondy, France; 3 Odum School of Ecology, University of Georgia, Athens, GA 30602, USA; 4 Center for Tropical and Emerging Global Diseases, University of Georgia, Athens, GA 30602, USA; 5 Center for the Study of Complex Systems, University of Michigan, Ann Arbor, MI 48109 USA; 6 Fogarty International Center, National Institutes of Health, Bethesda, MD 20892, USA

## Abstract

**Background:**

Influenza A viruses exhibit complex epidemiological patterns in a number of mammalian and avian hosts. Understanding transmission of these viruses necessitates taking into account their evolution, which represents a challenge for developing mathematical models. This is because the phrasing of multi-strain systems in terms of traditional compartmental ODE models either requires simplifying assumptions to be made that overlook important evolutionary processes, or leads to complex dynamical systems that are too cumbersome to analyse.

**Results:**

Here, we develop an Individual-Based Model (IBM) in order to address simultaneously the ecology, epidemiology and evolution of strain-polymorphic pathogens, using Influenza A viruses as an illustrative example.

**Conclusions:**

We carry out careful validation of our IBM against comparable mathematical models to demonstrate the robustness of our algorithm and the sound basis for this novel framework. We discuss how this new approach can give critical insights in the study of influenza evolution.

## 1 Background

The ecology and evolution of influenza viruses, especially in human and avian populations, have received considerable attention over the last decade [1-3]. It is increasingly recognised that because of the genetic diversity of these RNA viruses [4,5] and their high mutation rate [1], transmission dynamics and evolution need to be considered simultaneously. The evolutionary dynamics of influenza viruses are complicated the frequent occurrence of reassortment events [6,7], whereby two virus strains co-infecting the same individual exchange genetic material, resulting in a third strain. Despite its demonstrated frequent occurrence [1,6], reassortment remains largely absent from theoretical studies of influenza evolution. In this respect, it is important to develop an appropriate theoretical framework to explore its evolutionary impact.

The most commonly used approach to analyse influenza dynamics has been mathematical models where state space is expanded to take into account multiple virus strains [8,2,3,10]. The overlap between epidemiological and evolutionary time scales [1], the observed immune drift in human populations [2,3], and the high mutation rate [1] make it essential to integrate a large strain space into models. Consequently, the classic modelling approaches, based on the familiar *SIR *framework [11-13], are not readily amenable for this purpose because the resulting state space increases exponentially with the number of strains and, consequently, becomes too cumbersome for meaningful analysis. One possibility, recently suggested [14,3], is not to track co-infections, yielding a model whose number of state variables increase linearly with the number of strains. However, these models by necessity overlook the contribution of reassortment events (but see [15]).

It is possible to fill this void by developing an Individual-Based Model (IBM, see [16,17]). The rationale of this kind of modelling is to explore individual heterogeneity, allowing us to track infection history. Extensively used in ecology [18] and more recently in epidemiology [9], the main goal of these models have been to focus on very detailed and specific scenarios, with the goal of making quantitative predictions especially about alternative infection control decisions [19,20,9]. IBMs have also been shown to be useful in theoretical studies [21-23].

Here, we develop a stochastic IBM to simultaneously address the ecology and evolution of avian influenza viruses. We first describe the current paradigms of influenza mathematical modelling, which drives the structure of our computational framework. After detailing our IBM, we demonstrate that under analogous conditions, our model precisely recaptures the output of classic models. This validation step will allow the future use of our IBM to rely on the background of mathematical epidemiology [11-13]. Finally, we conclude that our model improves the current mathematical models and can be exploited to study evolutionary processes previously intractable, giving the opportunity to address all the components of influenza evolution.

### 1.1 Transmission process and mean field theory

The goal our of IBM is to extend the capabilities of the mathematical models of Influenza viruses. Here, we describe the current paradigms about the Influenza transmission and how they are modelled using the classic *SIR *framework. These models underlie the IBM design presented during the next section.

In humans populations, a direct transmission is assumed between individuals as usual when airborne propagation is documented [24]. The situation is more complex in birds. Generally, direct transmission is assumed to encapsulate both fecal/oral and airborne transmission because they are occur on the same time scale and both rely on the proximity of susceptible and infectious individuals [25]. There is increasing evidence for indirect influenza transmission in avian species through an environmental reservoir [26-29]. Experimental studies have shown the long-term persistence of these viruses in aquatic environments [4,30] and theoretical studies have underlined its importance in epidemiological and evolutionary outcomes [29,25,31,32].

Epidemiological models of influenza viruses generally consider only a limited number of strains, perhaps as few as two [33,31]. The host population is then classified according to their infectious status with regards to each strain. For a two strain model, for example, individuals are born susceptible to both strains (*N_SS_*) and become infected (*N_IS _*or *N_SI _*) at rate *λ_i_*. While infected with strain *i*, they may be exposed to strain *j *resulting in co-infection (*N_II _*), at a rate (1 - *σ*) *λ_j _*where *σ *captures the extent of cross-protection. Individuals recover (*N_RS _*or *N_SR_*) at rate *γ_i _*and are assumed to be invulnerable to that strain.

Clearly, the force of infection (*λ_i_*) is the main driver for epidemiological and evolutionary dynamics. Direct transmission (through airborne droplets and/or via the short-term fecal/oral route) is usually modeled as a density-dependent process [24] and defined by the rate *β_i_I_i_S *(*I*_1 _= *N_IS _*+ *N_II _*+ *N_IR _*and *I*_2 _= *N_SI _*+ *N_II _*+ *N_RI _*). For avian influenza viruses, the force of infection includes another component, intended to capture transmission via the environmental reservoir: , where *ρ *is the uptake rate, *L *is the lake volume, *κ *is the viral load needed to yield a 50% probability of infection, and *ε *is the competition term for viral particles within environment [34,33,29]. This competition term *ε *determines which strain is the most able to infect the susceptible individual. The viral load determining the infection rate, *V_i_*, is influenced by the shedding rate of infectious individuals *ω_i _*and the pathogen clearance rate in environment *ξ_i_*, also termed environmental durability. The mathematical formulation is:(1)(2)(3)(4)(5)(6)(7)(8)(9)(10)(11)(12)(13)(14)

When a single disease system is assumed, we can derive an analytic expression for the R_0_, a predominant measure in epidemiology measuring the expected number of infections when one infectious individual is introduced in a population totally susceptible:(15)

where  and  are the contribution of direct and environmental transmission respectively [25]. When evolution is considered, *i.e*. through mutation, this framework can be extended easily to numerous strains if full cross-immunity is assumed (*i.e*. *σ *= 0 and co-infection is not possible). However, partial cross-immunity is well established for influenza viruses [3,35]. In this case, the state space of the system increases exponentially because the infection history of individuals needs to be tracked, quickly leading to model intractability. A solution recently suggested [14] is to assume "polar immunity", which means that cross-immunity makes some of the hosts totally immune instead of infecting them. An infection is hence possible only if the host is completely susceptible to this strain. Then, it is possible to track only susceptible and infectious individuals to each strain *i*:(16)(17)(18)

where *σ_ij _*represents the cross-immunity network between strains, *m *is the mutation rate and *i *and *j *are the strain "identities" of the pathogens. Here, we assume that cross-immunity decreases exponentially [14] with the distance between the infection history and the challenging strain (equation 19) as it has been described for influenza viruses in humans [3] and horses [35]. This exponential decrease is shaped by the parameter *d *.

These ordinary differential equations may be converted to exact Markovian analogues using, for example, Gillespie's direct method [36,37,13]. To do so, first the rates of all events have to be specified (*i.e *transitions between classes, see table S1 in additional file 1). The time until the next events is computed at each iteration as follows:(19)

where *R_j _*is the rate of event *j *and *RAND*1 is a uniform random deviate in (0, 1). Then, a new uniform random number in (0,1) is generated and multiplied by ∑*^n ^R_j_*, denoted by *P *. The next event is then determined by ∑^*j*-1 ^<*P *< ∑*^j ^*.

## 2 Implementation

The recent development in mathematical models of influenza viruses fails to follow co-infections and consequently to incorporate reassortment. We choose the framework of IBM (Individual-Based Model, see [22]) to develop a model fully considering epidemiology and evolution of Influenza viruses.

### 2.1 Model structure

As usual in IBM, we use an oriented-object approach [38] where a "class" is an abstract pattern of a physical entity (*e.g*. a pathogen or a host) and an "object" an instance of its class (*e.g*. each host or each pathogen). Each class has its own "attributes" which represent the properties of an object. These attributes can be model parameters (*e.g*. host lifespan) and filled by the user (table 1). Otherwise, they change through time like state variables (table 2) and make links between classes (*e.g*. a "Host" object may contain several "Pathogen" objects). These variables are modified through "methods", which correspond to the different functions applicable to each object (*e.g*. pathogen transmission).

**Table 1:** Parameters of the model

Name	Class in IBM	Units	Value used in this study
*μ*	BIRD	years^-1^	2
*ρ*	BIRD	centiliter.day^-1^	10^4^
*L*	LAKE	centiliter	10^4^
*κ*	PATHOGEN	virions	10^2^
*ξ*	PATHOGEN	days^-1^	30
*β*	PATHOGEN	year^-1^	0.0078
*m*	PATHOGEN	ind.days^-1^	0.048
*γ*	PATHOGEN	days^-1^	7
*ω*	PATHOGEN	virions.day^-1^	10^12^
*d*	MODEL	None	5
*δ_t_*	MODEL	days	None
tMax	MODEL	day	None

Each parameters are explained in the main text. Values displayed here are used throughout the manuscript except something different is noticed.

**Table 2:** Variables of the model

Name	Class	Type	Description
currentPathogens	BIRD	PATHOGEN[]	Array with every strains in Infectious state

next Pathogens	BIRD	PATHOGEN[]	Array with every strains just enter in Infectious state

oldPathogens	BIRD	PATHOGEN[]	Array with infection history

Pathogens	LAKE	PATHOGEN[]	Array containing every strains present in the environment

ViralLoad	LAKE	INTEGER[]	Viral load of every stains present in the lake

identity	PATHOGEN	double	Strain identity

hostTab	MODEL	HOST[]	Array with all "Host" objects

Every of these attributes change dynamically through time and represent the environment, the host population and its infectious status

**Figure 1:**
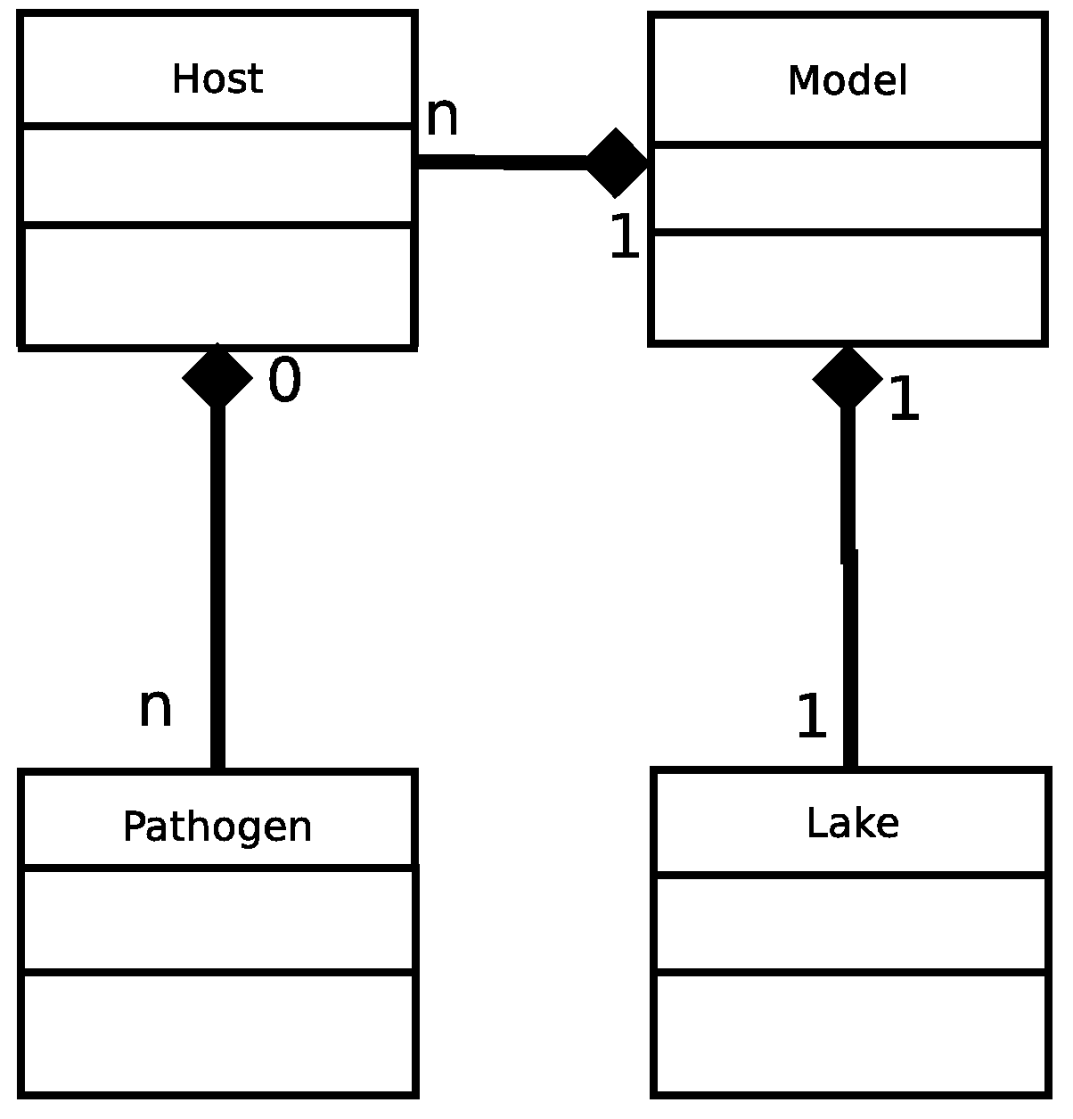
**UML design of the IBM model**. The links between classes represent a "composition" property. The filled diamonds points the containing class and the other side is the contained class. The "cardinalities" (1,n) represents the number of contained objects or the number of objects which can contain it. The methods and attributes of each class are detailed in the main text and are not displayed here for clarity.

The structure of our model, *i.e*. the relationships among classes, is described in Figure 1. This UML (Unified Modeling Language) design shows the different parts of our framework and how they are linked together. The instance of class "Model" represents the program scheduler and contains all "Host" objects corresponding to individuals. These last ones can become infected and entertain one or more memory reference to "Pathogen" instances. "Host" objects can also interact with the environment (class "Lake") by viral particles shedding through a method of "Model" object.

### 2.2 Dynamical Model behaviour

For the sake of simplicity, here we do not explain every functions in detail, but focus instead on the most important ones (all algorithms are detailed in the section S1 in additional file 1). Specifically, we explain the functions where infection, mutation or recovery events are applied. The probability of these events are calculated by applying an exponential distribution on rates, as usual in mathematical epidemiology (*P *= 1 - *exp*(- *rate *× *δ*_t_) where *δ*_t _is the model integration time step and *rate *is the individual rate expressed in the mean field approximation [13]).

#### 2.2.1 Program scheduler and host status

In IBM, two steps are generally considered at each iteration [39]. The first one determines the status of each individual at the end of this time step. Then, when all individuals have computed their next status, they are updated through a second function. This methodology is applied to avoid synchronization problems which can occur where an individual calculates its status based on other individuals status who have one more time step.

In our case, at each iteration, a function "Step" is called for every individual. If a host becomes infected to one specific strain during this function, a "Host" attribute ("nextPathogens") will be filled with a memory reference to this "Pathogen" object. After all individuals have called the "Step" function, the "Update" function moves this reference to another variable ("currentPathogens"). During the following time step, this reference can move to another variable ("oldPathogens") with a probability *P *= 1 - *exp*(- *γδ_t_*).

#### 2.2.2 Infection process

Within the "Step" function, transmission process (direct and/or environmental) takes place. For each strain present in the system at this time step, a probability of infection is computed for both transmission routes. In the case of direct transmission, this probability is equal to *P_i _*= (1 - *σ*)(1 - *exp*(- *βI_i_δ_t_*)). This probability is added to  which describes the environmental transmission (parameters have the meaning than in equations 1-13).

The mathematical formulation assumes that co-infections occur sequentially and not simultaneously. In fact, co-infections can be approximately simultaneous because of the very small time step involved in Gillespie's method. Time step duration is not infinitesimal in the IBM. To avoid an under-estimation of co-infections, we have to consider that co-infections occur sequentially, but potentially during the same time step. Then, we apply a method already used in [40]. We rank randomly infection probabilities for each individual and apply the transition if selected (if *RAND*1() <*P*). Ranking randomly the infections events removes the potential issue that pathogens at the top of the pathogen list can have a greater chance to infect the host.

#### 2.2.3 Mutations

Upon infection, mutations occurs at a fixed rate. At the end of the host update, a function "mutation" is called for every "Pathogen" objects found in the list "currentPathogens". The probability that a mutation occurs is given by *P *= 1 - *exp*(- *mδ*_t_). When this event is selected, the "identity" will increase or decrease by 1 with a probability of 50% each. Hence, we assume a linear strain space. Other pathogen parameters stay identical (*i.e. *no evolution of life-history traits).

#### 2.2.4 Demography

When all individuals have completed their "Update" function, host demography is applied. Hosts have an expected period before producing one o spring as well as an average lifespan. Each host produces one off spring (susceptible) with a probability *P *= 1 - *exp*(- *μδ*_t_) and dies with the same probability (assuming a constant population size).

#### 2.2.5 Viral demography within environment

To model the fluctuations of environmental viral concentration, we use the deterministic solution of the equations 10-11) as it has been calculated in [32]:(20)

### 2.3 Input and Output Files

The attributes which have to filled by the user are split between three different input files in order to distinguish their class destination. The "main" input file contains the global parameters of the simulation, *i.e. *the length of a time step, the lake volume or the filenames of other input files. The "species" file describes the ecology of the population considered. It integrates its birth, death and drinking rate, its population size and which transmission route has to be included. Finally, the "pathogen" file depicts the life-history traits of the introduced pathogens, *i.e. *its mutation and direct-transmission rate, the viral load required to yield 50% of infections, as well as its initial conditions within the host population (identity, infectious population size and environmental concentration). Finally, we have implemented three different outputs which track the pathogen dynamics at the environmental (dynamics of strains concentration), individual (all the infection events) and populational level (dynamics of strain infectious population size).

## 3 Results and discussion

### 3.1 Methodology for IBM validation

We compare IBM outputs in specific cases which can be addressed by the stochastic version of the two mathematical frameworks previously exposed (equations 1-13, see also table S1 in additional file 1). The goal of this validation step is to show that our algorithms reproduce correctly the expected behaviour of *SIR *framework.

Within the existing epidemiological studies using an IBM, a time step of one day is generally used and claimed to be small enough. To assess this, we analyse a first simple epidemiological case where only one strain and direct transmission are involved. These results determine the optimal time step duration to produce similar disease dynamics than in *SIR*. It is worth to point out that the period between two time steps has to be as small as possible to get the correct dynamics and large enough to reduce significantly the simulation time.

Then, we study a situation where two identical strains and environmental transmission are introduced. We compare the IBM outputs with the stochastic version of the *SIR *system depicted in equations 1-13. As usual when stochastic outputs are compared [13], we analyse a bunch of pathogen dynamics characteristics. For both strains, we analyse (i) the epidemics peak (in terms of infectious population size), (ii) the time at epidemics peak (time needed to reach the epidemics peak), (iii) the epidemic duration (time between the start and the end of the epidemics) and (iv) the sampled epidemics size (sum of all new infections during an epidemics). We study also the global properties of the epidemics: (v) the global extinction proportion (no epidemics are observed for both pathogens) and (vi) the ratio between dominance of strain 1 and dominance of strain 2 (epidemics occurs only for strain 1 over epidemics occurs only for strain 2).

Finally, we compare the evolutionary dynamics of our model with the model assuming polarity immunity (described in equations 17-18). We study this dynamics without environmental transmission since the analysis of its influence on evolutionary dynamics is beyond the scope of this paper.

### 3.2 Dynamical behaviour

With only direct transmission and one strain (*β*_2 _= ω_2 _= 0), we explore here the influence of the IBM time step duration in order to find its optimal value

**Figure 2:**
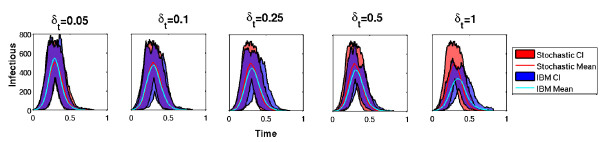
**Influence of time step duration involved in Individual-Based Model when only one strain and direct-transmission are assumed**. The blue shaded area and line show the IBM dynamics. The red shaded area and line shows the *SIR *dynamics. For the sake of clarity, only simulations without extinctions are considered among the 500 replications. Parameters are detailed in table 1.

Our simulations show that a time step of one day yields a too long and too flat disease dynamics comparing to *SIR *models (Figure 2). Even if the cumulative incidence (total number of infectious individuals during the epidemics) should be relatively similar, the discrepancy between disease dynamics may have some undesirable effects. Our results underlines that a time step of 0.1 day is enough to have similar dynamics between IBM and *SIR *models. This value will be used in the rest of this study.

### 3.3 Comparison of epidemiological signatures

We now compare IBM and the stochastic *SIR *outputs where two identical strains and environmental transmission are involved. Here, our goal is to show that our model yields epidemiological dynamics indistinguishable from the *SIR *model depicted in equations 1-13.

**Figure 3:**
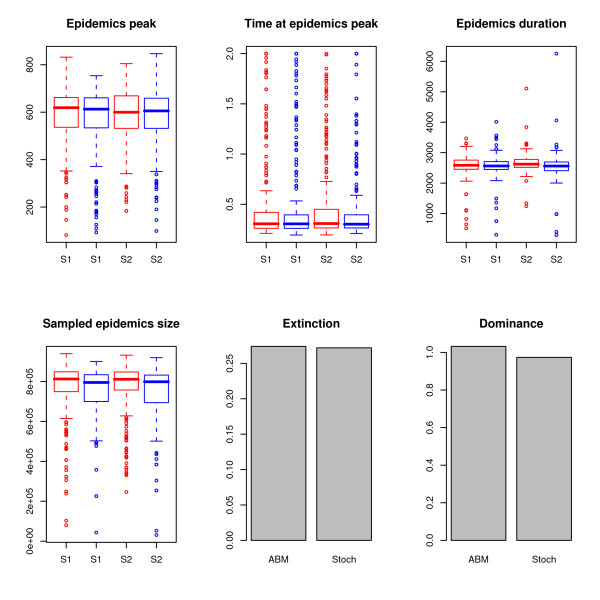
**Epidemiological validation of the IBM with two strains and environmental transmission**. The blue and red boxplots show the distributions of epidemiological signatures of IBM and *SIR *respectively. For the sake of clarity, these distributions have been computed only where at least one strain displays an epidemics (defined as an epidemic peak greater than 50 individuals). 500 replications have been used. The epidemiological signatures are described in the main text and the parameters used are detailed in table 1.

For both approaches, Figure 3 shows the distributions of the different epidemiological signatures for each strains and the proportions of global extinction and dominance ratio. We apply Kolmogorov-Smirnov and *χ*^2 ^tests to explore statistical difference between the distributions generated by the two models. All these tests produce *p *- *values *> 5% (see table S2 in additional file 1) and highlight that the differences between the epidemiological dynamics produced by our IBM and by *SIR *models do not reach an acceptable level of certainty. Hence, these distributions are statistically indistinguishable.

### 3.4 Evolutionary dynamics

We now focus on the adequacy between evolutionary dynamics produced by our IBM and by stochastic *SIR *model when only density-dependent transmission is involved (described in equations 17-18, implemented as in [3]). We assume the same cross-immunity pattern than in equation 19.

**Figure 4:**
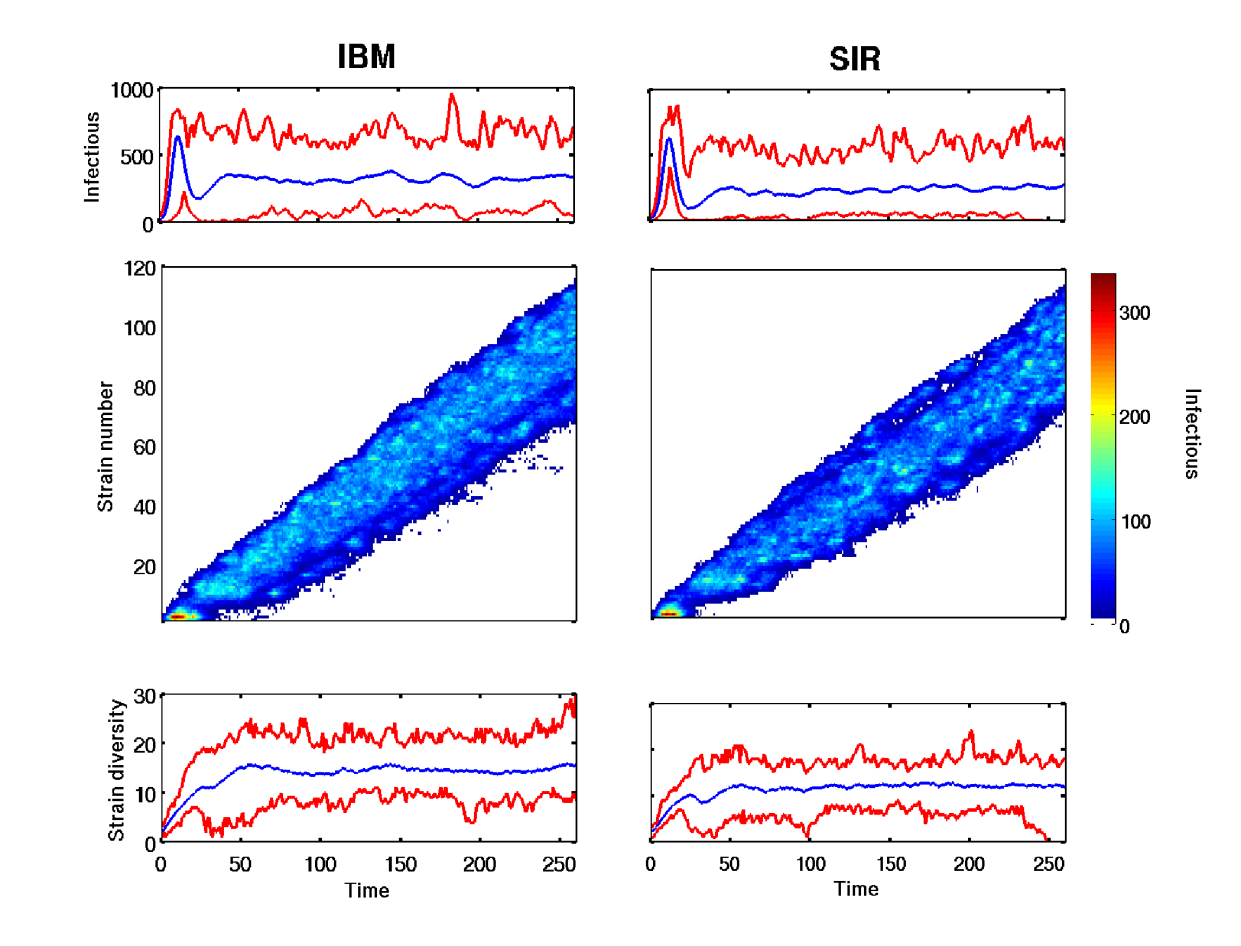
**Evolutionary dynamics of stochastic *SIR *model and IBM where only direct transmission is involved**. The top panels show the dynamics of the infectious individuals (summing all the strains through time). The middle panels show the evolutionary dynamics. Y-axis represents the strain "identity", X-axis is time and colors illustrate the superposed evolutionary dynamics of the 500 stochastic realizations. Each point in this panel is the maximal incidence over all these replicates for the strain identity described on Y-axis at the time step given on X-axis. The bottom panels show the dynamics of strain diversity. The left and right parts show the outputs of our IBM and *SIR *model respectively.

For the same mutation rate *m *and cross-immunity shape *d*, IBM and *SIR *models show similar evolutionary dynamics (Figure 4). The epidemiological dynamics are similar (top panels), despite that *SIR *model exhibits a lower prevalence. It is well known that this model, assuming polarity immunity, tends to underestimate the infectious population size [14]. The speed of cluster replacement is the same (medium panels) and, because of the under-estimation of infectious population size, the strain diversity through time (bottom panels) is slightly lower.

## 4 Conclusions & Discussions

In this paper, we have introduced a stochastic Individual-Based Model (IBM) to study epidemiological and evolutionary dynamics of avian influenza viruses. We have shown that we can accurately reproduce the solutions of classic *SIR *model as a special case of our model. However, the more general set of conditions that may be represented by the IBM will enable investigation of a much larger class of evolutionary and ecological dynamics.

Additional epidemiological signatures could be analysed. Nevertheless, our goal was to give the proof that our algorithms have been implemented correctly in order to can rely on *SIR *literature for the future improvements of our IBM. Since six different epidemiological signatures, tackling different aspects of the disease dynamics (coexistence, extinction, epidemics peaks, etc...), have been used (Figure 3), we believe that the overall picture allows us to claim that our IBM mimics *SIR *models with a computational formulation at an individual scale.

The main difference between IBM and *SIR *models is the possibility to acquire sequentially several infections within a same time step. We had to make this assumption since the period between two time steps is necessarily bigger in the IBM than in the mathematical framework where the time step is infinitesimal. Results reported here show that, despite this difference, the two formulations are dynamically identical.

We suggest that the discrepancy between the evolutionary dynamics produced by *SIR *model and by our IBM (Figure 4) is due to the known underestimation of the infectious population size consecutive to the assumption of polarity immunity [14]. Even if it is impossible to quantify this under-estimation, it is worth to point out that this dichotomy cannot be due to a time step too coarse in our IBM. Indeed, a too large IBM time step tends to underestimate infectious dynamics (as shown in Figure 3), instead of the opposite.

The possibility to track co-infections has been central in the design of this model, but it is valuable to underline that our IBM improves the current mean field theories in an additional way. Our model can cope with a strain space that is virtually infinite (constrained by the maximal number of a "double" variable, usually more than 10^300^). Hence, it is not necessary to invoke pragmatic approximations in order to simplify the model; the full epidemiological and evolutionary dynamics can be explored in our framework.

This IBM can be extended in numerous ways. Excepting all the possible computational additions (space, network interactions, etc..), complex ecological and evolutionary processes can also be included. So far, we have considered a constant host population size, but the modification of the demography function can integrate all kind of dynamics, even data from the field. Similarly, the reassortment process, which has never been theoretically studied as we said before, has not been exposed here because its analysis is beyond the scope of this paper. Nevertheless, it can be included easily through the mutation function. The strain identity should be replaced by a set of characters, representing amino acids for instance. Then, this string can be also divided into different parts to mimic the possibility of different genes. This modification of pathogen genome makes possible the creation of a third "Pathogen" object produced by the exchange of amino acids between strains co-infecting a given individual.

Here, we propose an Individual-Based Model improving the current modelling paradigms on influenza viruses. Its validation against a *SIR *model allows the future uses to rely on the *SIR *background. Its ability to be extended opens many new areas of influenza research which were previously constrained by the limitations of the mathematical formulation. To conclude, we shown that modelling at an individual scale allows the study of mathematically inaccessible situations despite an identical behavior. We believe this model offers an unique opportunity to fully address the evolution of influenza viruses.

## 5 Availability and requirements

Project name: InfluenzaIbm

Project home page: https://sites.google.com/site/rocheben/influenzaibm

Operating system(s): Platform independent

Programming language: C++

Other requirements: None

License: GNU GPL

## 6 Authors' contributions

BR participated to study conception, carried out the model programming, carried out the analysis of the model and drafted the manuscript. JMD participated to study conception and improved the manuscript. PR participated to study conception, model analysis and improved the manuscript. All authors read and approved the final manuscript.

## Supplementary Material

Additional file 1 **Appendix**. This file contains the algorithms used in this study, the description of the deterministic and stochastic *SIR *models and the results of the statistical tests. Click here for file
