# Supplementary material for: An Agent-Based Model to study the epidemiological and evolutionary dynamics of Influenza viruses
Source: BMC Bioinformatics. 2011 Mar 30;12:87. doi: 10.1186/1471-2105-12-87 (PMC3078862; doi:10.1186/1471-2105-12-87)
Supplement: Additional file 1 — Appendix. This file contains the algorithms used in this study, the description of the deterministic and stochastic SIR models and the results of the statistical tests. [file 1471-2105-12-87-S1.PDF]

An Agent-Based Model to study the epidemiological and evolutionary dynamics of Influenza viruses: Supplementary materials

January 28, 2011

Benjamin Roche[1,2], John M. Drake[3,4] and Pejman Rohani[1,5,6]

[1]Department of Ecology and Evolutionary Biology, University of Michigan, Ann Arbor, MI 48109, USA
[2]UMI IRD/UPMC 209 - UMMISCO, 93143, Bondy, France
[3]Odum School of Ecology, University of Georgia, Athens, GA 30602, USA
[4]Center for Tropical and Emerging Global Diseases, University of Georgia, Athens, GA 30602, USA
[5]Center for the Study of Complex Systems, University of Michigan, Ann Arbor, MI 48109 USA
[6]Fogarty International Center, National Institutes of Health, Bethesda, MD 20892, USA

S1 Algorithms

Signs // corresponds to a commentary. Algorithms presented here can differ from the code implemented to make it more readable.

S1.1 Class Model

```
VOID GO()
  BEGIN
    FOR (double t=0;t<tMax;t+=timeStep)
      currentTime=t;
      allDataWrite();
      writeLakeData();
      //Call step function
      FOR(int lIndex=0;lIndex<hostList.length();lIndex++)
        hostList.getElement(lIndex).Step();
      END FOR
      //Set the number of infectious individuals to zero for every strain
      infectiousInd.removeAllPathogens();
      //Call update function
      FOR(int lIndex=0;lIndex<hostList.length();lIndex++)
        hostList.getElement(lIndex).Update();
      END FOR
      //Update lake status
      theLake− >update();

      //Birth and death events
      List lAdd;
      List lRemove;

      FOR(int lIndex=0;lIndex<agentListPerso− >length();lIndex++)
        Host* lHost=hostList.getElement(lIndex);
        float lProba=1-exp(-(1/lHost.getbirthRate())×timeStep);
        IF(rand()<lProba)
          lAdd.add(lHost);
        END IF
        lProba=1-exp(-(1/lHost.getdeathRate())×timeStep);
        IF(randomObject− >getUnif()<lProba)
          lRemove.add(lAgent);
        END IF
      END FOR
      FOR(int lIndex=0;lIndex<lAdd.length();lIndex++)
        addHost(lAdd.getElement(lIndex));
      END FOR
      FOR (int lIndex=0;lIndex<lRemove.length();lIndex++)
        delHost(lRemove.getElement(lIndex));
```

```
        END FOR
    END FOR
END
```

S1.2 Class Host

```
// Computing cross-immunity level
VOID GETCROSSIMMUNITY(Pathogen* pPathogen)
    BEGIN
    float lReturn=0;
    IF(nextPathogen.length()==0 AND currentPathogen.length()==0 AND oldPathogen.length()==0)
        lReturn=1;
    ELSE
        double lSum=pPathogen.getSum();
        double lSumMax=pPathogen.getsumMax();
        double lMinDistance=lSumMax;
        FOR(int i=0;i<nextPathogen.length();i++)
            double lSumNew=nextPathogen.getElement(i).getSum();
            int lDistance=absValue(lSum-lSumNew);

            IF (lMinDistance>(lDistance))
                lMinDistance=lDistance;
            END IF
        END FOR
        FOR (int i=0;i<currentPathogen.length();i++)
            double lSumNew=currentPathogen.getElement(i).getSum();
            int lDistance=absValue(lSum-lSumNew);

            IF(lMinDistance>(lDistance))
                lMinDistance=lDistance;
            END IF
        END FOR
        //Looking for the closest pathogen in Recovered state
        FOR (int i=0;i<oldPathogen.length();i++)
            double lSumNew=oldPathogen.getElement(i).getSum();
            int lDistance=absValue(lSum-lSumNew);

            IF (lMinDistance>(lDistance))
                lMinDistance=lDistance;
            END IF
        END FOR
        //From equation 19 in the main text
        lReturn=model− >getmaxCrossImmunity()×(1-exp(-pow(lMinDistance/1,2)));
    END IF
```

```
    return lReturn;
END

VOID MUTATION()
BEGIN
    FOR(int lIndex=0;lIndex<currentPathogen.length();lIndex++)
        Pathogen* pPathogen=(Pathogen*)currentPathogen.getElement(lIndex);
        Pathogen* lPathogenTemp=new Pathogen(pPathogen);
        lPathogenTemp.mutation();
        Pathogen* lPathogen=model.getPathogenAdd(lPathogenTemp);
        delete lPathogenTemp;
        currentPathogen.setElement(lIndex,lPathogen);
    END FOR
END

VOID STEP()
BEGIN
    // Environmental transmission
    infectionWater();
    // Inter-individuals transmission
    infectionDirect();
END

VOID UPDATE()
BEGIN
    List toEliminate;
    //From S to I
    FOR (int i=0;i<nextPathogen.length();i++)
        toEliminate.add(nextPathogen.getElement(i));
    END FOR
    IF (toEliminate.length()>0)
        currentPathogen.addAll(toEliminate);
        nextPathogen.removeAll(toEliminate);
        toEliminate.removeAll();
    END IF
    //From I to R
    FOR(int i=0;i<currentPathogen.length();i++)
        float lrecoveryPeriod=currentPathogen.getElement(i).getrecoveryPeriod();
        float lProbaRecovery=1-exp(-(1/lrecoveryPeriod)×model.gettimeStep());
        IF(random().getUnif()<lProbaRecovery)
            toEliminate.add(currentPathogen.getElement(i));
        END IF
    END FOR
    IF(toEliminate.length()>0)
        oldPathogen.addAll(toEliminate);
        currentPathogen.removeAll(toEliminate);
```

```
        toEliminate.removeAll();
    END IF
    mutation();
END

VOID infectionWater()
BEGIN
    IF(drinkingRate!=0)
        List lPathog;
        List lProbaInf;
        lake− >getProbabilityInfection(drinkingVolume,lPathog,lProbaInf);

        List lIndexList;
        FOR(int lIndex=0;lIndex<lPathog.length();lIndex++)
            float *lIndexTemp=new float;
            *lIndexTemp=lIndex;
            lIndexList.add(lIndexTemp);
        END FOR
        FOR(int lIndex=0;lIndex<lPathog.length();lIndex++)
            float lIndexRand=round(model− >getRandom()− >getUnif()×(lIndexList.length()-1));
            float* lIndexRand1=(float*)lIndexList.getElement(lIndexRand);
            int lIndexTemp=(int)(*lIndexRand1);
            lIndexList.remove(lIndexRand1);
            delete lIndexRand1;

            float lCI=getCrossImmunity((Pathogen*)lPathog.getElement(lIndexTemp));
            float lProba=(*(float*)lProbaInf.getElement(lIndexTemp))×lCI;
            IF(random()<lProba)
                Pathogen* lTemp=(Pathogen*)lPathog.getElement(lIndexTemp);
                Pathogen* lPathogen=addPathogen(lTemp,lCI,true);
                IF(lPathogen!=0)
                    lPathogen− >setwaterInfect(true);
                    model− >dataWrite(indexSpecies,lPathogen);
                    lake− >addPathogen(lPathogen,0);
                END IF
            END IF
        END FOR
        FOR(int lIndex=0;lIndex<lProbaInf.length();lIndex++)
            delete (float*)lProbaInf.getElement(lIndex);
        END FOR
    END IF
END

//Function for inter-individual transmission
VOID infectionDirect()
BEGIN
```

```
    IF(contactRate!=0)
        List lPathog;
        ListlViralLoad;

        model.getNbInfect(lPathog,lViralLoad);

        List lIndexList;
        float* lIndexTempnew=new float[lPathog.length()];
        FOR(int lIndex=0;lIndex<lPathog.length();lIndex++)
            lIndexTempnew[lIndex]=lIndex;
            lIndexList.add(lIndexTempnew[lIndex]);
        END FOR

        FOR(int lIndex=0;lIndex<lPathog.length();lIndex++)
            double lUnif=model− >getRandom()− >getUnif();
            double lLength=(lIndexList.length()-1);
            float lIndexRand=round(lUnif×lLength);

            float* lIndexRand1=(float*)lIndexList.getElement((int)lIndexRand);
            int lIndexTemp=(int)(*lIndexRand1);
            lIndexList.remove((void*)lIndexRand1);

            Pathogen* lTemp=(Pathogen*)lPathog.getElement(lIndexTemp);
            float lTempNbInf=(*(float*)lViralLoad.getElement(lIndexTemp));

            float lProbaTemp=1-exp(-((lTemp− >getprobInfection()/365)×lTempNbInf)×model− >gettimeStep());
            float lCI=getCrossImmunity(lTemp);
            float lProba=lCI×lProbaTemp;
            IF(model− >getRandom()− >getUnif()<lProba)
                Pathogen* lPathogen=addPathogen(lTemp,lCI,true);
                IF(lPathogen!=0)
                    lPathogen− >setwaterInfect(false);
                    model− >dataWrite(indexSpecies,lPathogen);
                    lake− >addPathogen(lPathogen,0);
                END IF
            END IF
        END FOR
        delete [] lIndexTempnew;
    END IF
END

VOID AddPathogen(Pathogen* pPathogen,float pCI)
BEGIN
    Pathogen* lPathogenTemp=new Pathogen(pPathogen);
    Pathogen* lPathogen=model− >getPathogenAdd(lPathogenTemp);
    delete lPathogenTemp;
```

```
    nextPathogen.add(lPathogen);
END
```

S1.3 Class Lake

```
//Return pathogens drunk in the lake regarding the drinking volume and lake volume*/
LIST getProbabilityInfection(float drinkingVolume)
BEGIN
    List lProba;
    double lSum=0.0;
    FOR(int i=0;i<pathogens.length();i++)
        lSum+=*(float*)viralLoad− >getElement(i);
    END FOR
    FOR(int i=0;i<pathogens− >length();i++)
        Pathogen* lTempPath=pathogens.getElement(i);
        float* lTemp=new float;
        float lV=(*(float*)viralLoad− >getElement(i));
        float lKappa=lTempPath− >getviralLoadNeeded();
        //From equation 12 and 13 in the main text
        float lRate=( ( (float)drinkingVolume/365)/lakeVolume)×(lV/lSum)×(lV/(lV+lKappa));
        *lTemp=1-exp(-lRate×model− >gettimeStep());
        IF(lTemp>0)
            lProba.add(lTemp);
        END IF
    END FOR
END

//Apply viral demography
VOID viralDemography()
BEGIN
    List lListNbInf;
    lListNbInf=model.getNbInfect();
    //For each pathogens in lake
    FOR(int i=0;i<pathogens− >length();i++)
        //We get the pathogen and its viral load
        Pathogen* lPathogen=pathogens.getElement(i);
        float lViralLoad=viralLoad.getElement(i);
        float lTempInf=0;
        //We are looking for every infectious individuals
        FOR(int j=0;(j<(lPathog.length()) AND (lTempInf==0));j++)
            Pathogen* lPathogen2=lPathog.getElement(j);
            IF(lPathogen2− >samePathogenSequence(lPathogen))
                //If sequence is found, get the infectious population size
                lTempInf=(*(float*)lListNbInf.getElement(j));
            END IF
```

```
        END FOR
        float omega=(lPathogen.getexcretionVolume()×365);
        float I1=lTempInf;
        float xi=365/lPathogen.getlifespan();
        float V1=lViralLoad;
        float pT=model.gettimeStep()/365;
        // From equation 21 in the main text
        float lTemp=omega×I1/xi+exp(-xi×pT)×(V1-(omega×(I1)/xi));
        viralLoad.setElement(i,lTemp);
    END FOR
  END

  //Update viral load
VOID update()
  BEGIN
    viralDemography();
  END

  // Add a pathogen to the lake
VOID addPathogen(Pathogen* pPathogen,float pViralLoad)
  BEGIN
    bool lFound=false;
    FOR (int i=0;i<pathogens− >length();i++)
        IF(pPathogen− >samePathogenSequence( ((Pathogen*)pathogens− >getElement(i))))
            float lTempPointeur=viralLoad− >getElement(i)+pViralLoad;
            viralLoad− >setElement(i,lTempPointeur);
            lFound=true;
            break;
        END IF
    END FOR
    IF(lFound==FALSE)
        float* lTemp=new float;
        *lTemp=pViralLoad;
        pathogens− >add(pPathogen);
        viralLoad− >add(lTemp);
    END IF
  END
```

S1.4 Class Pathogen

```
// mutation process for pathogen
VOID mutation()
  BEGIN
    IF(model.getRandom().getUnif()<mutationRate)
        IF(sum==0)
```

```
            sum++;
        ELSE
            IF(model.getRandom().getUnif()<0.5)
                sum++;
            ELSE
                sum–;
            END IF
        END IF
    END IF
END
```

S2 Stochastic SIR model

The stochastic SIR model used for validation purposes is based on a two-strains SIR model with environmental transmission. The population size N is categorized into compartments regarding their infectious status. Rates of events are displayed below

Rate	Units	Transition
μ N	individual.years^{-1}	SS=SS+1
μ SS	individual.years^{-1}	SS=SS+1
μ SI	individual.years^{-1}	SI=SI+1
μ SR	individual.years^{-1}	SR=SR+1
μ IS	individual.years^{-1}	IS=IS+1
μ RS	individual.years^{-1}	RS=RS+1
μ RI	individual.years^{-1}	RI=RI+1
μ IR	individual.years^{-1}	IR=IR+1
μ II	individual.years^{-1}	II=II+1
μ RR	individual.years^{-1}	RR=RR+1
λ_1 SS	individual.years^{-1}	SS=SS-1, IS=IS+1
λ_2 SS	individual.years^{-1}	SS=SS-1, SI=SI+1
$\tau\lambda_1$ SI	individual.years^{-1}	SI=SI-1, II=II+1
$\tau\lambda_1$ SR	individual.years^{-1}	SR=SR-1, IR=IR+1
$\tau\lambda_2$ IS	individual.years^{-1}	IS=IS-1, II=II+1
$\tau\lambda_2$ RS	individual.years^{-1}	RS=RS-1, RI=RI+1
γ IS	individual.years^{-1}	IS=IS-1, RS=RS+1
γ SI	individual.years^{-1}	SI=SI-1, SR=SR+1
γ II	individual.years^{-1}	II=II-1, RI=RI+1
γ II	individual.years^{-1}	II=II-1, IR=IR+1
γ RI	individual.years^{-1}	RI=RI-1, RR=RR+1
γ IR	individual.years^{-1}	IR=IR-1, RR=RR+1

Table S-1: Rates and transition for each events in the stochastic SIR model.

S3 Test of significantly different distributions/proportions for IBM validation

Epidemiological signature and strain considered	P-value
Strain1/Epidemic peak	0.6725
Strain2/Epidemic peak	0.9059
Strain1/Time at epidemic peak	0.6161
Strain2/Time at epidemic peak	0.6079
Strain1/Epidemics size	0.06842
Strain2/Epidemics size	0.2858
Strain1/Epidemics duration	0.4689
Strain2/Epidemics duration	0.09696
Extinction	0.9576
Dominance of strain 1	0.8567
Dominance of strain 2	0.7717

Table S-2: Statistical test showing no significant difference in epidemiological signatures distributions. Kolmogorov-Smirnov test has been performed on all epidemiological signatures expecting extinction and strains dominance where the proportions have been test by a χ^2 test.
